# Les violences conjugales à Dakar

**DOI:** 10.11604/pamj.2015.22.182.6441

**Published:** 2015-10-22

**Authors:** Mohamed Maniboliot Soumah, Abdoul Wahab Issa, Mor Ndiaye, El Hadj Oumar Ndoye, Mamadou Lamine Sow

**Affiliations:** 1 Service de Médecine Légale et Médecine du Travail, Faculté de Médecine, de Pharmacie et d'Odontologie, Université Cheikh Anta Diop, Dakar, Sénégal

**Keywords:** Violence, conjugale, agression, victimes, Violence, domestic, aggression, victims

## Abstract

L'objectif était d’évaluer les aspects épidémiologiques des violences conjugales, identifier les facteurs de risques et les différents types de violences conjugales, évaluer les conséquences des violences conjugales sur la santé des victimes, afin d'améliorer la prise en charge des victimes et la prévention du phénomène. Il s'est agit d'une étude transversale effectuée de décembre 2012 à janvier 2013 à Dakar. Les données ont été recueillies, après consentement, sur fiche d'enquête anonyme soumise à toute personne volontaire vivant en couple et résidant à Dakar. L'analyse statistique a été effectuée avec le logiciel SPSS 13.0. Le nombre de personnes victimes de violences conjugales était de 60 soit 37,30% dont 31 femmes (51,70%) et 29 hommes (48,30%). Le sex-ratio était de 0,93. Parmi les victimes, 53 étaient scolarisées soit 88,30%. Le régime matrimonial était de type monogame dans 39 cas (65%) et polygame dans 21 cas (35%). La vie en couple durait depuis moins de 11 ans dans 60% des cas et durait de 11 ans à 20 ans au plus dans 26,6% des cas. L’étude des types de violences montrait la fréquence des agressions physiques. Les armes utilisées étaient surtout les armes naturelles. Les principaux facteurs de risque de violence conjugale sont les facteurs sociodémographiques, culturels et économiques comme le jeune âge, l'inégalité du genre, les jeunes couples, la précarité, le niveau d'instruction élevé. La prise en charge des victimes et la prévention du phénomène restent insuffisantes dans nos pays.

## Introduction

La lutte contre la violence envers les femmes d'une manière générale et contre la violence domestique en particulier préoccupe de plus en plus les organismes internationaux, nationaux et locaux. Cette lutte est reconnue comme une tâche d'intérêt public. Le Sénégal a ratifié la plupart des conventions internationales concernant la protection contre les violences basées sur le genre et adapté sa législation nationale à ce propos. Cependant, la lecture de la presse sénégalaise suggère une recrudescence des cas de violence sexuelle, d'agressions physiques ou de maltraitances basées sur le genre. Notre étude a pour objectifs d’évaluer les aspects épidémiologiques des violences conjugales, d'identifier les facteurs de risques, d'identifier les différents types de violences conjugales, d’évaluer les conséquences des violences conjugales sur la santé des victimes, afin d'améliorer la prise en charge des victimes et la prévention du phénomène.

## Méthodes

Notre étude s'est déroulée dans la ville de Dakar, capitale de la République du Sénégal. Il s'agit d'une étude transversale effectuée de décembre 2012 à janvier 2013. Nous avons établi deux questionnaires (un pour les femmes et un pour les hommes) respectant l'anonymat et contenant des questions fermées et ouvertes que nous avons soumis à toute personne volontaire vivant en couple et résidant à Dakar. Après consentement, une explication claire sur la manière de remplir les fiches d'enquête est donnée à la personne. Le questionnaire était administré et quand le niveau d'instruction ne le permettait pas, un enquêteur unique remplissait les différents items. Pour la collecte des données, nous avons choisi comme lieux l'hôpital Aristide le Dantec et l'hôpital Fann 2 centres hospitaliers nationaux (CHN) publics de niveau 3 de Dakar, qui compte 6 CHN. Nous avons par ailleurs consacré les week-ends et les jours fériés pour nous déplacer vers les ménages dans les différentes communes de la ville. Nous avons inclus toutes les personnes vivant en couple et résidant à Dakar ayant rempli correctement leurs fiches d'enquête. Nous avons exclu toutes les personnes ne vivant pas en couple, ne résidant pas à Dakar et celles ayant mal rempli ou n'ayant pas rempli leurs fiches d'enquête. Les critères recherchés étaient: l’âge, le lieu de résidence, la profession, le niveau d'instruction (primaire, secondaire, supérieur, aucun), le statut matrimonial (mariage, concubinage), le consentement au mariage, la durée de vie conjugale, l'option lors de l'union (monogamie, polygamie), l'existence d'une infertilité du couple, le nombre d'enfants (en distinguant filles et garçons), l'existence d'une grossesse, la pratique de la contraception et le recueil de l'avis du mari le cas échéant, l'existence de violences conjugales et leur type (menace, humiliation, chantage, insulte, violences physiques (coups de poing, baton, arme blanche, arme à feu, étranglement, projection d'objets, projection de la personne sur le mur ou sur le sol, autres), suspicion, répudiation, rapports sexuels forcés, marchandés ou refusés, impositions de pratiques ou positions vécues comme humiliantes, contrôle de l'emploi du temps, contrôle des dépenses et des revenus), la fréquence des violences conjugales, la présence de témoins lors des violences (enfants, ami(e)s, beaux-parents, public) le moment des violences (diurnes, nocturnes, jours ouvrables, week-end), les troubles secondaires aux violences (troubles émotionnels (colère, honte, sentiment de culpabilité ou d'humiliation, panique, anxiété, dépression), troubles psychosomatiques (digestifs, céphalées, palpitations, asthénie, troubles respiratoires, lombalgies, engourdissement, fourmillements, troubles des règles), troubles du sommeil (insomnie, cauchemars), troubles de l'alimentation (anorexie, boulimie), troubles cognitifs (difficulté de concentration et d'attention, amnésie, désorientation), troubles sexuels (dyspareunie, vaginisme, anorgasmie, troubles érectiles), abus de médicaments (antalgiques, antidépresseurs, anxiolytiques), de tabac, d'alcool, l'existence de plainte et l'autorité requise en la matière (police, juge, autorité traditionnelle), l'existence de consultation après les violences (psychologue, psychiatre, gynécologue, urgences, guérisseur, marabout, automédication, autres), les motifs de consultation, l'existence d'hospitalisations et leur durée, le facteurs liés au partenaire (age, profession, niveau d'instruction, l'abus de tabac, d'alcool, l'infidélité)). La participation à l'enquête a été volontaire et sans contrainte. Le choix de ne pas se soumettre au questionnaire a été respecté. Les données de l'enquête ont été collectées sur les fiches d'enquête et saisies sur un masque élaboré à partir du logiciel SPSS 13.0. L'analyse statistique a été effectuée avec le logiciel SPSS 13.0. L’étude descriptive avait permis de déterminer les fréquences, moyennes, intervalles de confiance et les écarts type. L’étude analytique avait permis de faire la comparaison des fréquences grâce aux tests du khi^2^ ou de Fisher.

## Résultats

Durant l'enquête, 161 personnes ont répondu au questionnaire. Nous comptions 85 femmes (52,80%) et 76 hommes (47,20%). Parmi elles, 159 étaient mariées (98,75%) et 2 vivaient en concubinage (1,25%). L’âge moyen était de 38,29 ans avec des extrêmes de 17 et 65 ans. Le nombre de personnes victimes de violences conjugales était de 60 (37,30%) dont 31 femmes (51,70%) et 29 hommes (48,30%). Le sex-ratio était de 0,93. L’âge moyen des victimes était de 38,47 ans avec des extrêmes de 23 et 64 ans. Les victimes habitaient le plus souvent dans les quartiers populaires (43 victimes). Les conjoints de victimes avaient un niveau d'instruction élevé (niveau post baccalauréat pour 41,70%). Parmi les victimes, 53 étaient scolarisées (88,30%). Cinquante neuf victimes étaient mariées (98,30%) et seule 1 victime vivait en concubinage. La vie en couple était consentie dans 55 cas (91,70%) et forcée dans 5 cas (8,30%). Le régime matrimonial était de type monogame dans 39 cas (65%) et polygame dans 21 cas (35%). Sur les 31 femmes victimes, 4 (12,90%) étaient en état de grossesse, 8 victimes étaient sous contraception (25,80%) dont 2 sans en informer leur mari. Sur l'ensemble des victimes, l'infertilité était retrouvée dans 2 cas (6,45%) et ne concernait que les hommes. La vie en couple durait depuis moins de 11 ans dans 60% des cas et durait entre 11 et 20 ans au plus dans 26,6% des cas. L’étude montre que les violences conjugales n’épargnent aucun couple avec ou sans enfant. Leur fréquence est beaucoup plus importante au sein des couples qui ont 1 ou 2 enfants avec une même proportion de 21,70% (p = 0,002). Elle est rare à partir de six enfants. Concernant le sexe, les couples ayant uniquement des garçons ou des filles sont plus exposés aux violences conjugales (p = 0,001). La fréquence est la même dans les deux cas de 75%. L’étude des types de violences montrait la fréquence des agressions physiques (Tableau 1). Les armes utilisées étaient surtout les armes naturelles (coups de poing, coup de pied) et les projectiles à portée de main de l'agresseur (Tableau 2). Les violences étaient répétées (41,70%) et se déroulaient devant des témoins dans 35 cas (58,30%). Ces témoins sont généralement les enfants, les parents, les amis et/ou le public. Les violences conjugales surviennent pendant la nuit dans 36 cas (60%), la journée dans 28 cas (46,70%) et les week-ends dans 17 cas (28,30%). Ces violences engendraient des conséquences psychologiques sur les victimes (Tableau 3). Après une violence conjugale, 14 victimes (23,30%) ont porté plainte dont 8 hommes et 6 femmes. Les victimes ayant consulté après une violence conjugale sont au nombre de 17 (28,30%) dont 12 femmes et 5 hommes. Les femmes victimes ont consulté aux urgences, un psychologue et un gynécologue dans deux cas chacun, un guérisseur traditionnel dans trois cas et un marabout dans sept cas. Quant aux hommes la consultation a eu lieu aux urgences, chez un psychiatre, chez un guérisseur traditionnel et chez un marabout dans deux cas chacun. Parmi les victimes ayant consulté, 4 parmi elles (2 hommes et 2 femmes) ont bénéficié d'un arrêt de travail dont la durée varie de 1 à 21 jours avec une moyenne de 9,75 jours.

**Tableau 1:** Répartition des victimes selon le type de violence conjugale

Type de violence	Nombre		Total	Pourcentage
	Homme	Femme		
Humiliation	5	13	18	30
Menace	6	13	19	31,70
Chantage	6	5	11	18,30
Insulte	9	8	17	28,30
Agression physique	12	13	25	41, 70
Suspicion	11	2	13	21,70
Contrôle du temps	8	13	21	35
Contrôle de l'argent	9	14	23	38,30
Rapports sexuels forcés	0	5	5	8,30
Rapports sexuels refusés	4	1	5	8,30
Marchandage de l'acte sexuel	2	0	2	3,30
Répudiation	0	3	3	5
Imposition de pratiques ou de positions vécues humiliantes	0	0	0	0

**Tableau 2:** Types d'agression physique et leur fréquence

Type d'agression	Nombre		Total	Pourcentage
	Homme	Femme		
Coups de poing	6	12	18	72
Bâton	2	0	2	8
Arme blanche	1	0	1	4
Arme à feu	1	0	1	4
Etranglement	0	3	3	12
Projection d'objet	3	3	6	24
Projection de la personne sur le sol	4	2	6	24
Projection de la personne contre le mur	1	4	5	20

**Tableau 3:** Fréquence des troubles engendrés par les violences conjugales

Troubles	Nombre		Total	Pourcentage
	Homme	Femme		
Psychologiques	17	25	42	70
Psychosomatiques	7	13	20	33,3
Sommeil	11	19	30	50
Alimentation	5	9	14	23,3
Cognitifs	6	13	19	31,7
Troubles sexuels	6	8	14	23,3
Abus de médicaments	0	5	5	8,3
Abus d'alcool	3	1	4	6,70
Abus de tabac	6	1	7	11,70

## Discussion

Ce travail est une étude préliminaire sur un sujet d'actualité. La difficulté majeure rencontrée était la réticence des populations interrogées avec dans la plupart des cas, remise de fiches vierges ou mal remplies malgré le consentement. Pour les personnes n'ayant pas consenti, « c'est leur vie privée dans laquelle nous n'avons pas droit de nous ingérer et le thème que nous abordons est un tabou au Sénégal ». Comparativement, les deux Boutiques de Droit installées dans les quartiers populaires de Dakar (Médina et Pikine) ont recensés 263 violences conjugales durant toute l'année 2014 (source ministère de la justice). Notre enquête sur 2 mois portant sur 161 personnes nous a permis de trouver 60 violences conjugales. Cela doit conduire à une enquête plus large telle l'Enquête Nationale sur les Violences Envers les Femmes en France. Les études réalisées en Tanzanie [1] et en France [2] concernaient des échantillons importants. La réticence des populations de Dakar face à un sujet tabou jusqu’à une époque récente, explique la faible participation à notre enquête. La violence conjugale est vécue dans le silence dans nos sociétés d'Afrique de l'ouest. L’étude montre qu'en matière de violences conjugales, aucun partenaire n'est épargné. Par rapport aux hommes, les femmes sont deux fois plus souvent agressées physiquement au sein du ménage, et trois fois plus souvent victimes d'attouchements ou de rapports sexuels forcés à l'extérieur comme à l'intérieur du ménage [3–6]. Les hommes sont également concernés par les violences conjugales. Néanmoins, la violence conjugale envers les hommes est moins fréquente, sans doute aussi encore moins visible que la violence conjugale envers la femme, pour des raisons sociales [7].

### Facteurs favorisants

La femme est dans de nombreuses sociétés africaines considérée comme un être inférieur à l'homme et donc sensée se soumettre à lui. Les normes sociales établissant des relations de pouvoir inégal entre les hommes et les femmes sont profondément ancrées dans ces sociétés. Elles déterminent et instaurent des inégalités flagrantes dans les rôles sociaux attendus entre les sexes. Ce modèle se transmet de génération en génération par le biais d'une éducation qui dès l'enfance est inculquée aux filles et aux garçons. Les petits garçons sont élevés dans le but de devenir des hommes virils et des pères de famille capables de tenir leur ménage en se faisant respecter. Les petites filles quant à elles se doivent d'apprendre à devenir des épouses obéissantes et des bonnes mères au foyer capables d'endurer des situations pénibles dans leur travail domestique et leurs maternités. En outre, le modèle conjugal s'impose clairement aux femmes comme le seul cadre de vie acceptable. En effet, le statut de femme autonome célibataire, c'est à dire s'assumant hors de toute tutelle masculine, est dans la plupart des sociétés d'Afrique subsaharienne considéré comme non digne de respect. Des influences diverses ont affecté les populations du Sénégal et leurs trois principales ethnies, à savoir les Wolofs, l'ensemble Peuls/Toucouleurs et les Sérères. Les coutumes et traditions entourant le mariage vont être influencées par les prescriptions du Coran avec l'islamisation progressive du pays, puis le colonisateur français tentera d'inscrire le mariage dans la laïcité à travers de nouvelles lois. La prégnance de la religion et des pratiques musulmanes dans la vie quotidienne des autochtones se manifeste dans la recherche d'un statut particulier, dès 1840, lorsque la décision est prise d'appliquer le code civil métropolitain dans la colonie du Sénégal. L’âge minimum au mariage « civil » est actuellement fixé à 16 ans pour les filles et 18 ans pour les garçons (code de la famille, 1990). Chrétiens et musulmans connaissent la même situation avec cette dichotomie associant modernité et traditions, union civile et union religieuse.

### Age

Dans notre étude, la violence conjugale concerne les sujets jeunes, ce qui concorde avec les données de la littérature [2, 6, 8]. On observe une diminution du taux global des violences conjugales avec l'accroissement de l’âge, reliée par les auteurs à la baisse des déclarations des violences et harcèlements psychologiques. Cela serait sûrement liée à un phénomène d’« *habituation*» des femmes vivant la situation depuis longtemps et ne considérant plus ces faits de harcèlement comme violents.

### Niveau scolaire

Parmi les victimes 53 sont scolarisées (88,30%) dont 25 (13 hommes et 12 femmes) de niveau supérieur. Selon Evelyne Josse, les femmes plus instruites sont exposées à un plus grand risque de violence et notamment de violence sexuelle de la part de leur partenaire intime. Parce qu'elles deviennent plus autonomes, elles résistent davantage aux normes patriarcales. Pour reprendre le contrôle, certains hommes recourent alors à la violence [9]. Par contre pour Mc Closkey L. et al., le niveau scolaire peu élevé de la femme (primaire) constitue un facteur de risque de violence conjugale [10]. François Pursell I. mentionne que les niveaux d’étude et les catégories socio-culturelles ne sont pas corrélés à la violence conjugale [7].

### Statut du couple

Dans les sociétés africaines le mariage forcé, la polygamie et l'infertilité sont considérés comme facteurs favorisants ou aggravants les violences conjugales. Ce qui n'est pas le cas de notre étude. Nos résultats se rapprochent de ceux d'Al Ilika et al., qui ont rapporté dans leur série 60% de femmes victimes de violences conjugales vivant dans un foyer monogame et 11% dans un foyer polygame [11]. Au Sénégal, comme dans de nombreux pays africains, l'Islam a sacralisé une institution ancienne comme la polygamie. Le législateur laïc sénégalais va manifester vis-à-vis de la question de la polygamie la même prudence que l'administration coloniale. La polygamie est reconnue, pour les musulmans, dans la législation moderne et le code sénégalais de la famille offre trois options matrimoniales: le régime de la monogamie, le régime de la limitation de la polygamie et le régime de la polygamie, auquel cas l'homme ne peut avoir simultanément plus de quatre épouses. L'option monogamique une fois signée est irrévocable pour toute l'existence de l'intéressé. Les seuls cas où la loi permet de revenir sur l'option de régime sont ceux dans lesquels le nouveau choix est destiné à le rendre plus restrictif par exemple lorsqu'on a choisi la polygamie limitée à trois épouses qu'on veut ramener à deux ou opter pour un régime monogamique. L’égalité des co-épouses, prônée par le Coran, est également soulignée dans le code qui stipule « *qu'en cas de polygamie, chaque épouse peut prétendre à l’égalité de traitement par rapport aux autres* ».

### Durée de la vie conjugale

Dans notre étude, la fréquence des violences conjugales est importante au sein des couples jeunes (de durée ≤10 ans) et régresse après 20 ans. Elle est respectivement de 61,70% et 11,70%. Le mariage en Afrique Noire était d'abord une union entre deux familles. Les époux engageaient à travers le mariage religieux et/ou coutumier, deux familles. Le couple habitait en général la concession familiale ce qui permettait une supervision par les parents, tel un apprentissage de la vie de couple. Ce n'est que plusieurs années plus tard que le jeune couple s'autonomisait en allant habiter hors de la concession familiale. Les transformations de la société africaine obligent les jeunes couples à vivre dans des appartements selon un modèle occidental, dès la célébration du mariage. Les crises traversées par le couple ne sont connues de l'entourage que lorsqu'elles sont graves, ayant franchi l’étape du passage à l'acte violent.

## Conclusion

Les principaux facteurs de risque de violence conjugale sont les facteurs sociodémographiques, culturels et économiques comme le jeune âge, l'inégalité du genre, les jeunes couples, la précarité, le niveau d'instruction élevé. Parmi les victimes hormis un couple qui vit en concubinage, tous les autres sont des mariés avec une nette prédominance des monogames. Le nombre d'enfants semble avoir une influence sur la survenue de la violence conjugale avec fréquence importante dans les foyers ayant un nombre d'enfants ne dépassant pas deux. L'agression, le contrôle d'argent, le contrôle du temps, la menace, l'humiliation et les insultes sont les types de violence les plus fréquents. Au cours des agressions les armes naturelles sont les plus utilisées. Les violences conjugales persistent et posent avec acuité un problème de santé publique. La prise en charge des victimes et la prévention du phénomène font défaut ou restent insuffisantes dans nos pays. Les autorités, les mouvements associatifs, les religieux doivent se mobiliser pour endiguer ces violences, dans le cadre général des violences. Le parcours des victimes de violences n'est pas uniforme sur le territoire. Les victimes ont accès à une consultation médico-judiciaire à l'Hôpital Général de Grand-Yoff, qui permet une meilleure caractérisation des lésions.
